# Anti‐Obesity Effects of *Leuconostoc mesenteroides* 4‐Fermented Lemon Peel Filtrate on HFD‐Induced Obese Mice via NFκB/ PPAR‐γ Pathway

**DOI:** 10.1002/fsn3.70039

**Published:** 2025-02-18

**Authors:** Xianrong Zhou, Yang Fan, Jia Liu, Ruokun Yi, Yongpeng He, Xin Zhao, Lujun Chen

**Affiliations:** ^1^ Chongqing Collaborative Innovation Center for Child Nutrition and Health Development Chongqing University of Education Chongqing People's Republic of China; ^2^ Chongqing Engineering Research Center of Functional Food Chongqing University of Education Chongqing People's Republic of China; ^3^ Chongqing Engineering Laboratory for Research and Development of Functional Food Chongqing University of Education Chongqing People's Republic of China; ^4^ Department of Bioscience Silla University Busan Republic of Korea; ^5^ Department of Cardiology The Second Affiliated Hospital of Chongqing Medical University Chongqing People's Republic of China; ^6^ Department of Clinical Nutrition Chongqing University Jiangjin Hospital Chongqing People's Republic of China; ^7^ Chongqing Key Laboratory of Translational Research for Cancer Metastasis and Individualized Treatment Chongqing University Cancer Hospital & Chongqing Cancer Institute & Chongqing Cancer Hospital Chongqing People's Republic of China; ^8^ Department of Pediatrics First Affiliated Hospital of Gannan Medical University Ganzhou Jiangxi People's Republic of China

**Keywords:** lemon peel, *Leuconostoc mesenteroides*, mice, NFκB/PPAR‐γ signaling pathway, obesity

## Abstract

Obesity is a major health problem associated with Type 2 diabetes, non‐alcohol fatty liver disease (NAFLD), and atherosclerosis. Functional lactic acid bacteria‐fermented products have been reported to have potential anti‐obese effect. The present results revealed that *Leuconostoc mesenteroides* 4 (LMSS4)‐fermented lemon peel filtrate slowed down the increase of body weight, and decreased liver and epididymal fat indices; it also decreased serum levels of TC (total cholesterol), TG (triglyceride), LDL‐C (low‐density lipoprotein cholesterol), ALT (alanine transaminase), AST (aspartate transaminase), and AKP (alkaline phosphatase), TNF‐α (tumor necrosis factor‐α), IFN‐γ (interferon gamma), IL‐1β (interleukin‐1β), IL‐6 (interleukin‐6), and IL‐10 (interleukin‐10), increased the levels of HDL‐C (high‐density lipoprotein cholesterol), IL‐4 (interleukin‐4), and IL‐10 (interleukin‐10). Furthermore, the mRNA expression of NFκB‐p65 (nuclear factor‐κB p65), PPAR‐γ (eroxisome proliferator‐activated receptor γ), TNF‐α, IL‐1β, leptin, SREBP‐1c (sterol regulatory element binding protein‐1c), FAS (fatty acid synthase), and CEBP/α (CCAAT/enhancer binding protein α) were down‐regulated, while the expression of IL‐4, IκB‐α (inhibitory subunit of NF Kappa B alpha), and IL‐10 were upregulated after the mice were treated with LMSS4‐fermented lemon filtrate; the filtrate also downregulated the protein expression of NFκB‐p65 and PPAR‐γ but increased the expression of IκB‐α. The HPLC results found that rutin and hesperidin were the predominant constituents in both the unfermented and LMSS4‐fermented lemon filtrates among the 15 constituents analyzed. In addition, chlorogenic acid, umbelliferone, byakangelicin, and oxypeucedanin hydrate were increased in the fermented lemon filtrate, in which chlorogenic acid showed the highest increase rate (83.51%). In conclusion, the anti‐obesity effect of LMSS 4‐fermented lemon peel filtrate was mediated via the regulation of the NFκB/PPAR‐γ signaling pathway. These results could provide an experimental basis for developing new functional lemon beverages for obesity intervention.

## Graphical abstract

LMSS4‐Fermented lemon peel filtrate can ameliorate high‐fat diet‐induced obesity in mice by modulating the NFκB/PPAR‐γ signaling pathway. HPLC analysis revealed that the levels of chlorogenic acid, umbelliferone, byakangelicin, and oxypeucedanin hydrate in fermented lemon peel filtrate were higher than those in unfermented lemon peel filtrate. The present study could provide data support for the application of fermented lemon products in weight loss beverages.

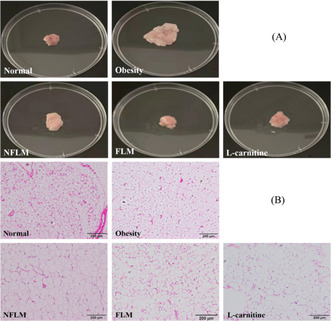

## 1 Introduction

Obesity is a global epidemic affecting nearly 2 billion people worldwide. It is predicted that the number of overweight and obese individuals will increase to 3.3 billion by 2030 (Koenen et al. 2021). Traditionally, the body mass index (BMI) is used to define obesity. Obesity is classified into different categories according to the World Health Organization criteria. Accordingly, BMI < 18.5 kg/m^2^ is defined as underweight, whereas 18.5–24.9 kg/m^2^ indicates normal weight and 25–29.9 kg/m^2^ is overweight. Obesity is also divided into classes I, II, and III based on BMI values of 30–34.9, 35–39.9, and ≥ 40 kg/m^2^, respectively (Mandic et al. 2023). The incidence of obesity is related to many factors, including unhealthy dietary habits, sedentariness, genetics, and environment, as well as endocrine and metabolic disorders (Yang et al. 2013). Obesity is always accompanied by other comorbidities, such as Type 2 diabetes, NFLD, atherosclerosis, dyslipidemia, and hypertension (Franks et al. 2010; Kim et al. 2018; Cote et al. 2013), which not only affect health but also increase the economic and medical burden of society. Therefore, it is essential to identify safe and effective methods to inhibit or delay the progression of obesity.

The fight against obesity involves both ordinary folks and expert researchers. Body weight can be controlled by decreasing the intake of high‐fat and high‐glucose diets and performing regular exercise. However, these two strategies appear to have little effect on decreasing the obesity levels in the population because of poor dietary discipline (Karachaliou et al. 2020). In addition, drugs and surgery are widely used to control severe obesity. Surgical interventions mainly include sleeve gastrectomy, gastric banding, and gastric bypass, which are associated with potential risks in obese patients who manifest other obesity‐related complications (Haley et al. 2015). Drugs for obesity intervention usually include orlistat, lorcaserin, and L‐carnitine. L‐carnitine is always selected as a positive drug in animal studies of obesity (Zhang et al. 2014; Wu et al. 2015). Although these drugs effectively inhibit obesity, their side effects include oily stools, diarrhea, flatulence, and decreased absorption of fat‐soluble vitamins (Khera et al. 2016).

Lemon, also called *Citrus limon* (L.) *Burm*. *f*., is a nutritionally and economically valuable fruit consumed worldwide. Nearly 2.330 million tons of lemon and limes are harvested every year in China, most of which are processed into juice, dried lemon slices, and lemonade. The by‐products of lemons and limes, such as peels, seeds, and pulps, contribute to the environmental burden (Liu et al. 2021). In addition to its beautiful appearance, pleasant smell, and good taste, the bioactivity of lemon is well established. Lemon is rich in d‐limonene, hemicellulose, carotenoids, polyphenols, dietary fibers, soluble sugars, flavonoids, cellulose, pectin, methane, ascorbic acid, and essential oils (Dosoky and Setzer 2018). Lemon exhibits anti‐oxidative, antibacterial, anti‐nausea, anti‐spasmodic, and anti‐inflammatory activities (Zhao et al. 2020). Therefore, studies investigating the bioactivity of lemon peels, seeds, and pulps are of great value.

The pathogenesis of obesity involves complex metabolic and inflammatory responses. In recent years, an increasing number of studies have demonstrated a close association between serum lipid indicators, liver function markers, and inflammatory indices with the occurrence of obesity. Elevated levels of TG, TC, and LDL‐C in serum are often observed alongside obesity, suggesting that obesity may lead to disturbances in lipid metabolism (Zhang et al. 2018). Furthermore, liver function markers (such as ALT, AST, and AKP) are frequently elevated in obese patients, reflecting the risk of associated conditions such as fatty liver disease (Wu et al. 2020). In terms of inflammatory response, obesity is often accompanied by a state of systemic inflammation, characterized by increased levels of pro‐inflammatory cytokines such as TNF‐α, IL‐6, and IFN‐γ, alongside a decrease in anti‐inflammatory cytokines such as IL‐4 and IL‐10. These inflammatory factors further contribute to insulin resistance, creating a vicious cycle (Sun et al. 2012). Concurrently, leptin, an important hormone secreted by adipocytes, is closely related to energy balance and appetite regulation, with levels often significantly elevated in obese individuals. Additionally, key transcription factors such as SREBP‐1c, FAS, and C/EBPα play vital roles in lipogenesis. Research indicates that the expression of these genes is markedly increased in obesity, further influencing lipid metabolism and insulin sensitivity (Kim et al. 2011).

Fermentation by lactic acid bacteria is an essential method used in food processing, which not only enhances the sensory properties, and extends the shelf‐life, but also improves the nutritional value of fruits and vegetables. It changes both the profile and types of bioactive compounds and converts phenolic compounds to molecules with added biological value (Septembre‐Malaterre et al. 2018). A previous study revealed that Lactobacillus‐fermented apple juice inhibited weight gain, decreased the lipid levels of TG, TC, and LDL‐C, and reduced the number of white adipose cells in high‐fat diet (HFD)‐induced obese mice (Han et al. 2021). Lemon peels contain sugars and a portion of lignin, which can be used as substrates for fermentation (Teigiserova et al. 2021). Some studies also found that lemon peels contain higher amounts of polyethoxylated flavones (notably tangeretin and nobiletin), flavanones (generally hesperidin and naringin), and phenolic compounds (p‐coumaric, primarily caffeic, sinapic acid and ferulic acids) compared with the edible part of the fruits (Singh et al. 2020). Fermented lemon peel decreases the inflammatory activity of LPS‐induced RAW264.7 cells (Kim et al. 2019). It prevents human dermal fibroblasts from UVA‐induced photoaging (Bae et al. 2012). Studies demonstrated that chronic intake of xylitol had no effect on glucose absorption in obese patients, and intake of xylitol may inhibit the development of obesity (Bordier et al. 2021). However, few studies investigated the anti‐obesity effects of fermented lemon peel. Studies exploring these effects may reduce environmental pollution, improve the utilization of lemon by‐products, and development of dietary beverages.

*Leuconostoc mesenteroides* 4 originated from Korean kimchi known as “Seokbakji”. The in vitro resistance of this strain at pH 3.0 artificial gastric acid was 81.35% ± 3.20% and 28.67% ± 0.93% in the presence of 3.0% bile salt, which suggests potential probiotic properties. In the present study, LMSS 4 was used to ferment the lemon peel. An obese mouse model was established to evaluate the anti‐obesity effect of LMSS 4‐fermented lemon peel filtrate.

## 2 Materials and Methods

### 2.1 Experimental Sample

*Leuconostoc mesenteroides* 4 (LMSS4), which originated from Korean kimchi known as “Seokbakji”, LMSS4 was provided by the Department of Food Science and Biotechnology of Cha University (Gyeonggi, South Korea), and the 16S rRNA sequence data of LMSS4 is provided in Attachment 1. Experimental lemons were purchased from the native market, this lemon genotypes were *Citrus* × *limon (Linnaeus) Osbeck*, situated in Tongnan District, Chongqing, China (30°26′28″ north, 105°31′41″ east, elevation 350 m above sea level, annual average temperature 17°C). Xylitol was ordered from Chongqing Maobai Technology Co. Ltd. (Chongqing, China).

### 2.2 Experimental Animals

Fifty‐sixty‐week‐old male SPF C57BL/6 mice were purchased from Chongqing Byrness Weil Biotech Ltd. [Chongqing, China, SCXK (XIANG) 2019–0004]. The mice were adaptively fed in a room under controlled temperature (25°C ± 2°C) and humidity (50% ± 5) and alternating 12 h light/12 h dark conditions. During this period, the mice were fed with standard mouse chow and water. All the experiments were conducted in accordance with the 2010/63/EU directive and the guidelines for the ethical review of laboratory animal welfare of the People's Republic of China (GB/T 35892‐2018) and institutional rules considering animal experiments.

### 2.3 Production of Lemon Peel Filtrate

The collected fresh lemon peel was pulverized with a plant tissue pulverizer (Ningbo Xinzhi Biotechnology Co. Ltd., Ningbo, China). The lemon peel sample, sterile distilled water, and xylitol were weighed in a triangular flask according to the material‐to‐liquid ratio of 1:30:3 (w/v/w). The LMSS 4 bacterial liquid (4%) was also added to the triangular flask. After thorough mixing, the triangular flask was transferred to a constant temperature air bath shaker (Thermo Fisher Scientific Inc., Waltham, MA, USA) for fermentation at 37°C, 0.29 × g, and 24 h. After the fermentation was completed, the fermentation broth was centrifuged at 9500 × g for 10 min. The supernatant was filtered with a 0.22 μm membrane filter. The filtrate was collected and stored in a 4°C refrigerator for later use. Unfermented lemon peel filtrate was treated the same as fermented lemon peel filtrate except that no lactic acid bacteria were added.

### 2.4 Obese Mouse Models

Fifty mice were randomly divided into five groups (*n* = 10/group): normal group, obese group, unfermented lemon peel filtrate group (NFLM group), fermented lemon peel filtrate group (FLM group), and L‐carnitine group. From Week 1 to 8, all mice except for the normal group mice were fed a 45% HFD. Mice in the normal group were fed a 10% standard fat diet. Additionally, from Week 5 to 8, obese mice received different treatments as follows: (1) Mice in the normal and obese groups were administered sterile distilled water orally at 9:00 a.m. every day; (2) mice in the NFLM group were orally administered unfermented lemon peel filtrate (0.1 mL/10 (g.bw)) at 9:00 a.m. every day; (3) mice in the FLM group were treated with oral LMSS 4‐fermented lemon peel filtrate (0.1 mL/10 (g.bw)) at 9:00 a.m. every day; and (4) mice in the L‐carnitine group were orally administered L‐carnitine (200 mg/(kg. BW)) at 9:00 a.m. every day. During the whole period, the body weights of mice were measured once a week.

### 2.5 Measurement of Liver and Epididymal Fat Index

All the mice were executed by spine dislocation. Liver and epididymal fat were collected and weighed. Using the following formula (1), the liver and epididymal fat index was calculated. organ index (%) = organ weight (g) × 100/body weight (g).

(1) $$\text{Organ index}\;\left(\%\right)=\frac{\text{organ weight}\;\left(g\right)}{\text{body weight}\;\left(g\right)}\times 100$$

### 2.6 Pathologic Examination

A soybean size of the liver and epididymal fat tissues were cut and placed in 10% paraformaldehyde solution and fixed for 2448 h. The tissue samples were embedded in paraffin, sectioned, and stained to observe the pathological changes under an electron microscope. Liver cells were stained with hematoxylin–eosin (H&E) and Oil‐Red O, and epididymal fat was only stained with hematoxylin–eosin (H&E).

### 2.7 Measurement of Serum Lipids and Liver Function Indicators

The collected whole mouse blood was centrifuged at 1500 × *g* and 4°C for 10 min. The upper serum was used to determine the levels of lipid indicators. The levels of TC, TG, LDL‐C, HDL‐C, ALT, AST, and AKP in mouse serum were determined according to the instructions provided in Nanjing JianCheng reagent test kits (Nanjing JianCheng Bioengineering Institute, Nanjing, China).

### 2.8 Elisa

The serum levels of TNF‐α, IFN‐γ, IL‐1β, IL‐4, IL‐6, and IL‐10 were measured via ELISA (Shanghai Enzyme‐Linked Biotechnology Co. Ltd., Shanghai, China).

### 2.9 RT‐qPCR Assay

According to the previous methods with slight modification (Chen et al. 2022a), the total RNA in mouse liver and epididymal fat were extracted with TRIzol reagent, followed by cDNA synthesis from RNA (1 μg) using the cDNA reverse transcription kit. Next, PCR amplification was performed under the following conditions: cDNA 1 μL, TaqMan Multiplex Master Mix 10 μL, 10 μM primer 2 μL, and ddH_2_O 7 μL. The amplification and detection were conducted in a real‐time fluorescence quantitative PCR instrument (Thermo Fisher Scientific Inc., Waltham, MA, USA). The amplification conditions were 95°C for 15 s, 55°C for 30 s, 72°C for 35 s, and the total number of cycles was 40. Finally, based on the CT value and formula 2^−ΔΔCT^ the relative gene expression was calculated, using β‐actin as the internal reference gene.

### 2.10 Western Blot

As described previously (Zhou et al. 2021), the Western blot was used to determine the obesity‐related protein expression in liver tissues. First, the Radio Immunoprecipitation Assay (RIPA) reagent plus phenylmethanesulfonyl fluoride (10000:1) was used to extract the liver protein. The BCA kit was used to quantify proteins. The protein samples were prepared using a protein‐loading buffer ratio of 2.5:1. A total protein sample of 50 μg was loaded to 10% SDS‐PAGE precast gels at 200 V for 45 min. Second, the separated protein bands were transferred from the gel to the polyvinylidene difluoride (PVDF) membrane (25 V, 3 min). The PVDF membrane was treated with 5% skim milk for 1 h at room temperature and then incubated with primary antibodies (NFκB‐p65, 5970‐MSM2‐P1, 1:100; IκB‐α, PA5‐118117, 1:1000; PPAR‐γ, A304‐460A‐M, 1:1000; β‐actin, PA1‐16889, 1:10,000; Thermo Fisher Scientific Inc., Waltham, MA, USA) for 2 h at 37°C and 0.087 × g. Third, the membrane was incubated with a secondary antibody (HRP, G‐21040, 1:10,000, Thermo Fisher Scientific Inc., Waltham, MA, USA) for 1 h after washing 5 times with PBS, 5 min each time. Finally, the protein bands were observed and recorded using Tiangen chemiluminescence imaging system (Tanon Science & Technology Co. Ltd., Shanghai, China) with a chemiluminescence solution (Beijing Solarbio Biological Technology Co. Ltd., Beijing, China).

### 2.11 HPLC Analysis of FLM and NFLM

A column from Agilent Poroshell ECC8 (i.e. 4.6 × 150 mm, 2.7 μm) was utilized in conjunction with an Agilent 1260 Infinity chromatography system that included a quaternary pump, an autosampler, a thermostatic column compartment, and a photodiode array detector (PDA). The solvent system comprised an aqueous solution of phosphoric acid at 0.025% (v/v) (A), methanol (B), acetonitrile (C), and a solvent mixture of water/acetonitrile/tetrahydrofuran in a volume ratio of 55/20/25 (D), with a flow rate set at 1 mL/min. The solvent program featured several isocratic periods: from 0 to 7 min, the composition was 63% A, 33% B, and 4% C; from 9 to 17 min, it changed to 57% A, 32% B, 5% C, and 6% D; at 19 min, the ratio adjusted to 47% A, 22% B, 5% C, and 26% D; at 29 min, it comprised 47% A, 8% B, 19% C, and 26% D; at 32 min, the mixture included 40% A, 0% B, 34% C, and 26% D; 35 min featured 33% A, 0% B, 41% C, and 26% D; and finally, from 40 to 45 min, it was set at 27% A, 0% B, 73% C, and 0% D. After 50 min, the solvents reverted to the initial conditions and were maintained for an additional 25 min to ensure column rebalancing, with the column temperature held at 30°C. The PDA was configured to scan wavelengths between 210 and 400 nm, operating at a sampling frequency of 2.5 Hz, while monitoring specifically at 330 nm. The complete ultraviolet (UV) absorbance spectra for each standard compound were obtained and compiled into a custom library. The identification of constituents involved comparing the full UV spectra and retention times of the sample peaks against those of the standards (Li et al. 2021).

### 2.12 Statistical Analysis

All the present data were analyzed using Excel (Microsoft, Redmond, USA) and GraphPad Prism 7.0 (Graph Pad Software, La Jolla, CA, USA). One‐way analysis of variance (ANOVA), followed by Duncan's multiple comparisons, was selected to analyze group differences. Results were expressed as mean ± standard deviation ($\bar{x}$ ± SD), and a *p* value < 0.05 was considered significant.

## 3 Results

### 3.1 Bodyweight Changes of Obese Mice

**FIGURE 1:**
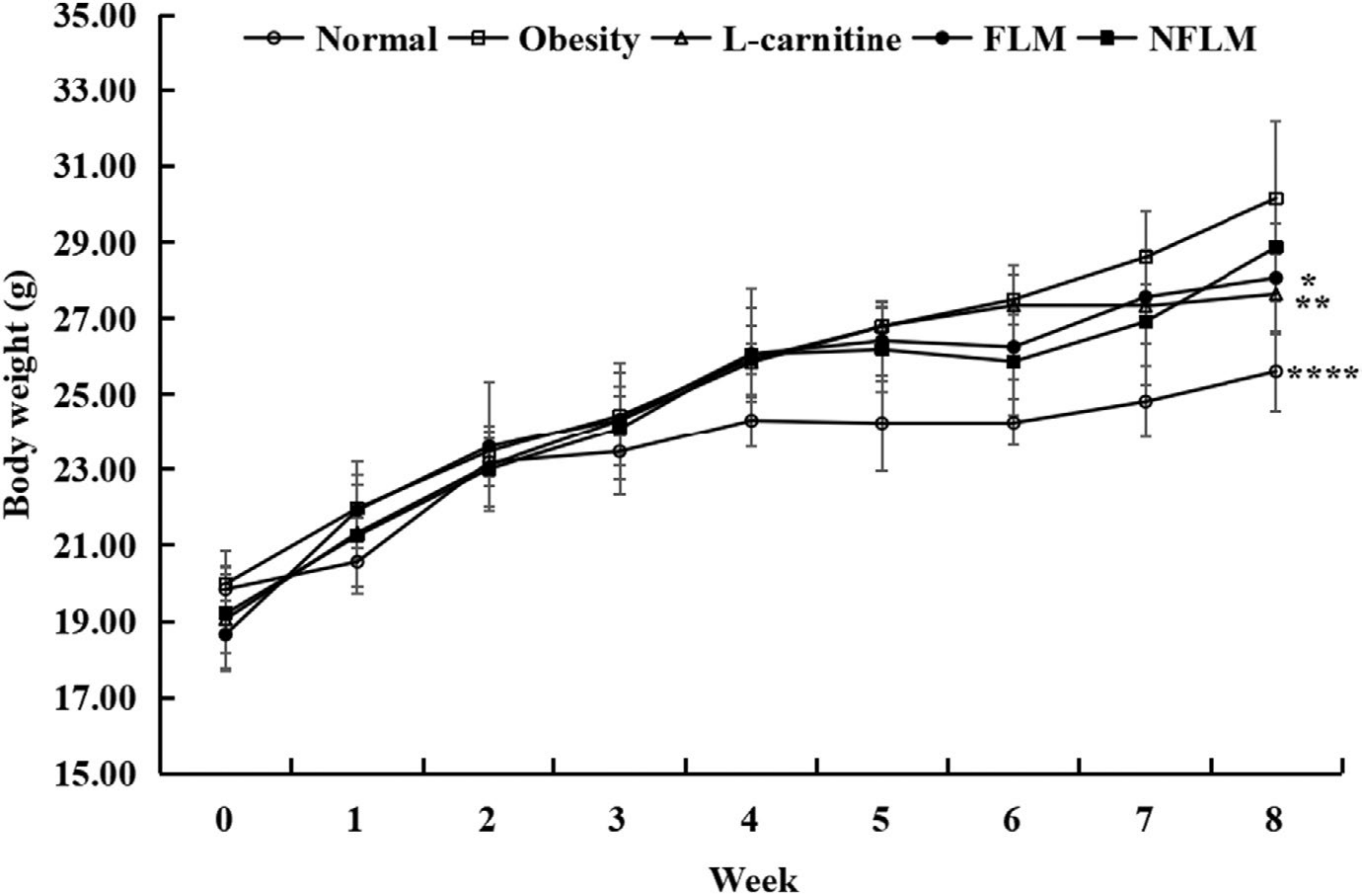
Bodyweight changes of obese mice. FLM, LMSS4‐fermented lemon peel filtrate; NFLM, non‐fermented lemon peel filtrate. Compared with Obesity group, *p < 0.5, **p < 0.01, ****p < 0.0001.

During the whole experiment, the body weight was measured once a week (Figure 1). The body weight in each group showed almost no difference in the first 3 weeks but changed obviously from Week 4. The mouse body weight in the normal group, which was fed with standard mouse chow, increased most slowly starting Week 4. However, although the mouse body weight showed no obvious differences between mice in different groups, the increase in body weight occurred in the following order: obese group > NFLM group > FLM group > L‐carnitine group.

### 3.2 Liver and Epididymal Fat Indices of Obese Mice

**FIGURE 2:**
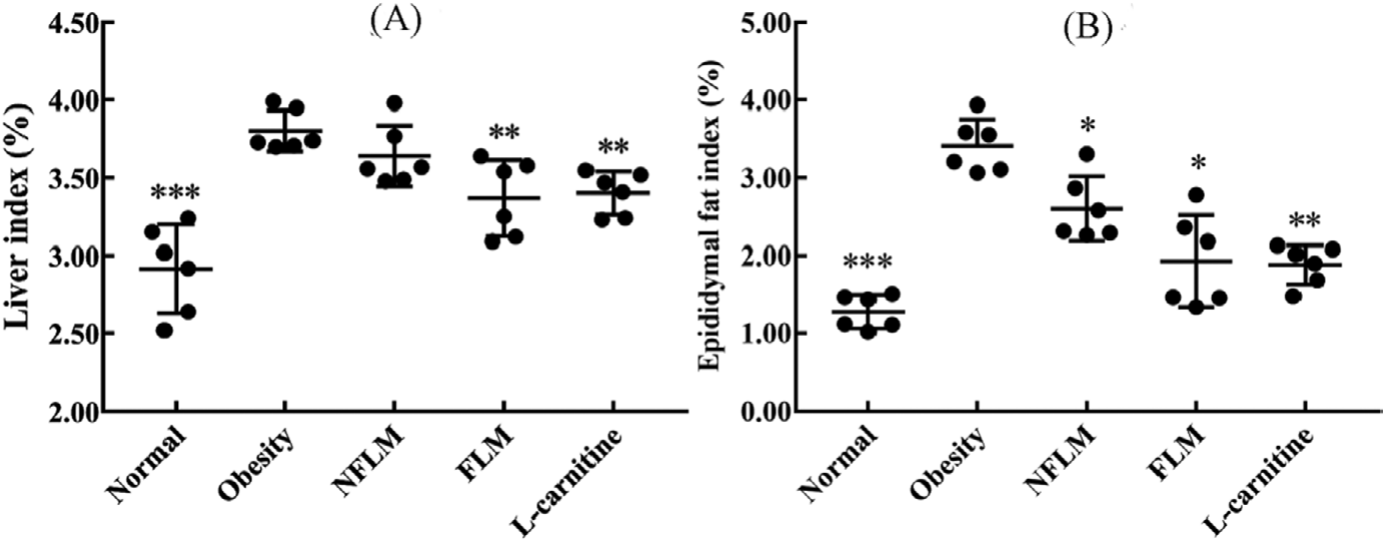
Liver and epididymal fat index of obese mice. (A) Liver index (%); (B) Epididymal fat index (%). NFLM, non‐fermented lemon peel filtrate group; FLM, LMSS4‐fermented lemon peel filtrate group. The data were expressed as mean ± standard deviation ($\bar{x}$ ± SD). Compared with Obesity group, *p < 0.5, **p < 0.01, ***p < 0.001.

Liver and epididymal fat indices are strong indicators of fat accumulation in obese mice. As shown in Figure 2, liver and epididymal fat indices were the least in the normal group. However, they were significantly higher in the obese mice than in the normal group. Oral administration of LMSS 4‐fermented lemon filtrate led to a significant decrease in the liver and epididymal fat indices in the obese group, and the effect was better than in the NFLM group and similar to the L‐carnitine group. Thus, LMSS 4‐fermented lemon filtrate inhibits fat accumulation in obese mice.

### 3.3 Liver Pathology

**FIGURE 3:**
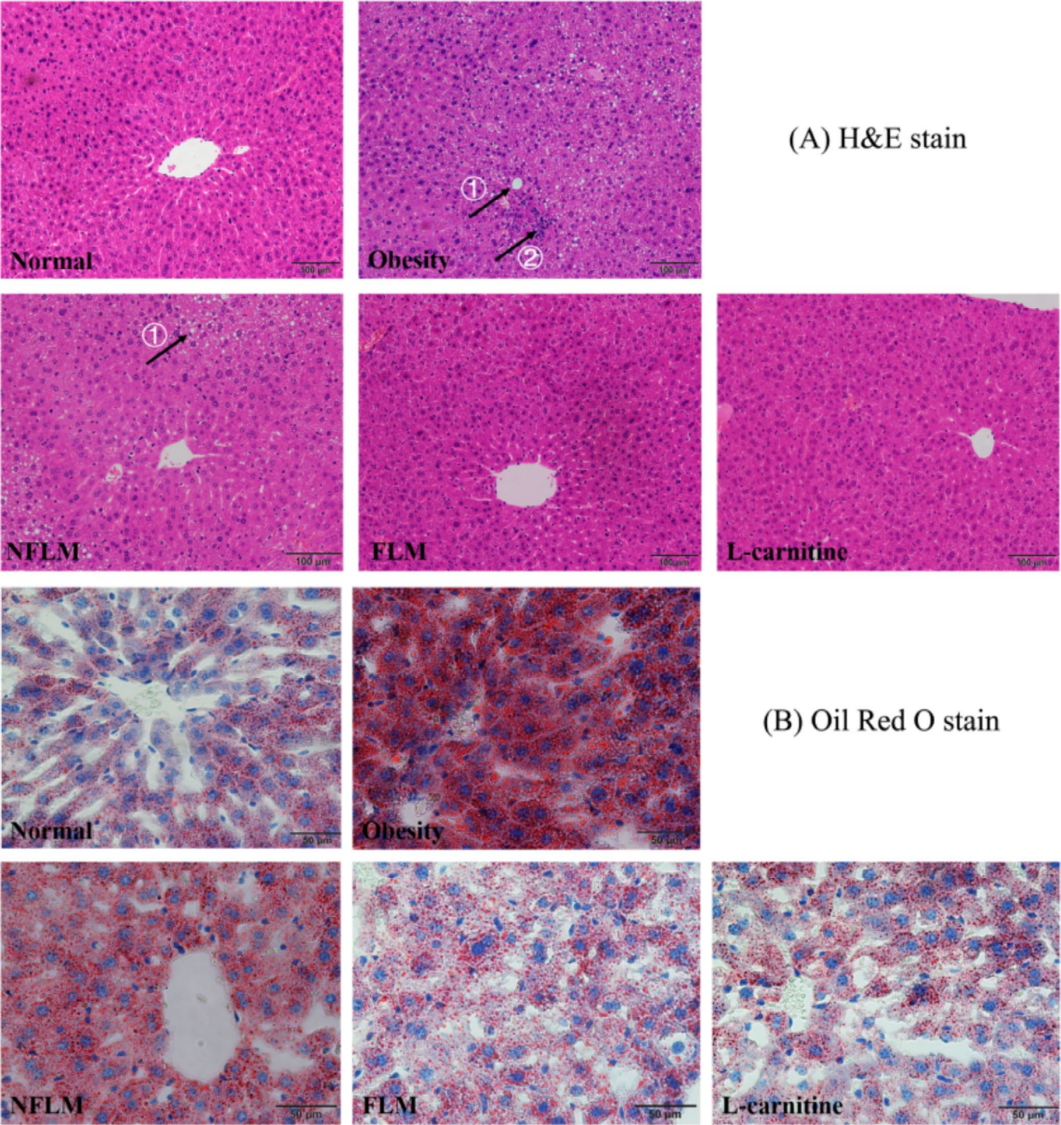
Liver pathologic observation of obese mice. (A) H&E staining of the liver; (B) Oil Red O staining of the liver. NFLM, non‐fermented lemon peel filtrate group; FLM, LMSS4‐fermented lemon peel filtrate group. The bars in (A) and (B) respectively represent 100 and 50 μm, and the arrows with “①” represent lipid droplets, and “②” represent necrotic cells.

Obesity induces fat accumulation in the liver. As shown in Figure 3A, the normal group showed intact liver structure with hepatocytes evenly distributed around the central vein in a satellite firing pattern. No necrotic cells were found. Conversely, the liver cell distribution was disorderly around the central vein. Multiple white lipid droplets in liver cells squeezed the nuclei in the liver cells to the edge of the cell membrane, resulting in necrosis. Compared with the obese group, the liver structure in the NFLM, FLM, and L‐carnitine groups was more intact. The lipid droplets also obviously decreased. The livers of mice in the FLM group were better than those in the NFLM group and similar to normal and L‐carnitine groups. Consistent with the results of H&E, the Oil Red O stained most parts of the mouse liver red in the obese group, suggesting fat accumulation. Compared with the obese group, the red color of the liver was obviously decreased in the NFLM, FLM, and L‐carnitine groups of mice, which suggested both lemon peel filtrate solution and L‐carnitine reduced the fat accumulation in the liver. However, the fat content in the FLM group was less than in the NFLM group but similar to the normal and L‐carnitine groups.

### 3.4 Appearance and Pathologic Observation of Epididymal Fat

**FIGURE 4:**
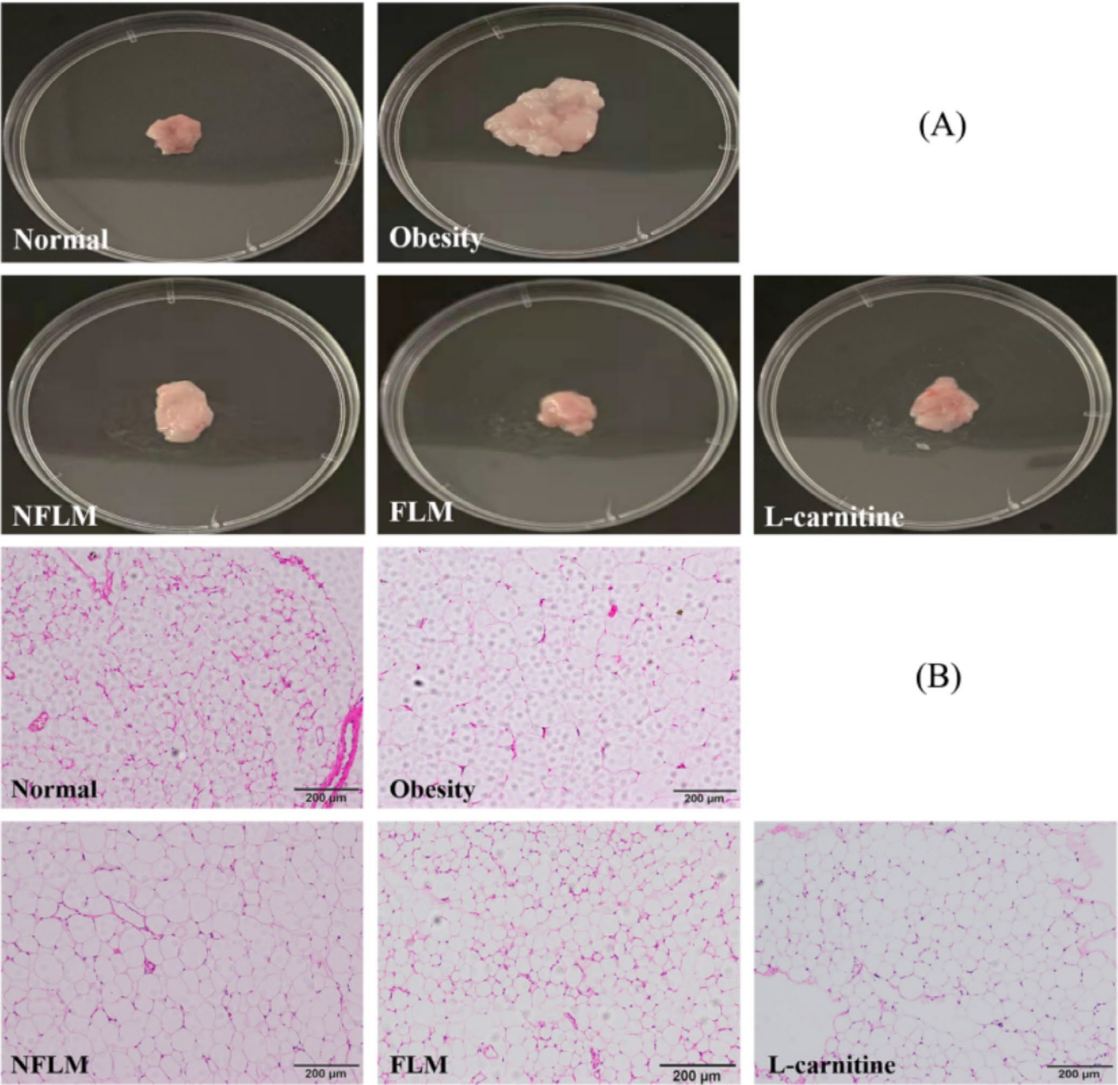
Appearance and pathologic observation of epididymal fat. (A) Appearance of epididymal fat; (B) H&E staining of epididymal fat. NFLM, non‐fermented lemon peel filtrate group; FLM, LMSS4‐fermented lemon peel filtrate group. The bars in (B) represent 200 μm.

As shown in Figure 4A,B, epididymal fat content was the largest and most distinct in the obese group, but the smallest and atrophied in the normal group. Compared with the obese group, the epididymal fat size decreased and cell numbers increased under the same magnification after treatment with unfermented and fermented lemon peel filtrate and L‐carnitine. The anti‐fat effect of fermented lemon peel filtrate was better than that of unfermented lemon peel filtrate and was similar to the normal and the L‐carnitine groups.

### 3.5 Serum Lipid Indicators of Obese Mice

As shown in Table 1, TG, TC, and LDL‐C levels were significantly higher in the obese group than in the normal group, while HDL‐C level was less than that of the normal group. Compared with the obese group, the serum TG, TC, and LDL‐C levels decreased, whereas HDL‐C levels increased after mice were treated with unfermented lemon peel filtrate, fermented lemon peel filtrate, and L‐carnitine. In addition, the effects of fermented lemon peel filtrate on serum lipids were better than those of unfermented lemon peel filtrate. The serum lipid levels in the FLM group of mice were similar to those in the normal and L‐carnitine groups, which indicates that fermented lemon peel filtrate maintains the serum lipid levels in a relatively normal condition.

**TABLE 1:** Serum lipid level of obesity mice.

Group	TG (mmol/L)	TC (mmol/L)	HDL‐C (mmol/L)	LDL‐C (mmol/L)
Normal	0.52 ± 0.04***	2.46 ± 0.09***	4.45 ± 0.36***	0.06 ± 0.01***
Obesity	0.82 ± 0.06	3.10 ± 0.17	2.92 ± 0.38	0.36 ± 0.08
NFLM	0.71 ± 0.06*	2.83 ± 0.05*	3.59 ± 0.10**	0.26 ± 0.04*
FLM	0.66 ± 0.12**	2.76 ± 0.18**	4.02 ± 0.23***	0.23 ± 0.04**
L‐carnitine	0.68 ± 0.02*	2.67 ± 0.17***	3.92 ± 0.39***	0.25 ± 0.06*

*Note:* Compared with Obesity group, *p < 0.5, **p < 0.01, ***p < 0.001.

### 3.6 Liver Function Indicators in Obese Mice

The serum levels of AST, ALT, and AKP reflect the degree of liver damage. As shown in Table 2, the ALT, AST, and AKP activities were the lowest in the normal group, while the highest activities were found in the obese group. Treatment with unfermented lemon peel filtrate, fermented lemon peel filtrate, and L‐carnitine led to a decrease in ASL, AST, and AKP levels, and these indicators in the FLM group and the L‐carnitine group were significantly lower than in the obese group.

**TABLE 2:** Hepatic function indicators of obesity mice.

Group	ALT(U/L)	AST(U/L)	AKP(King unit/100 mL)
Normal	226.26 ± 11.27***	19.50 ± 2.25***	6.37 ± 0.91***
Obesity	266.37 ± 7.27	37.28 ± 5.02	10.22 ± 1.40
NFLM	257.04 ± 4.17	32.75 ± 4.81	8.58 ± 1.21*
FLM	246.97 ± 3.50**	29.96 ± 4.16*	7.99 ± 0.99**
L‐carnitine	243.78 ± 3.81***	30.21 ± 3.35*	7.92 ± 0.58**

*Note:* Compared with Obesity group, *p < 0.5, **p < 0.01, ***p < 0.001.

### 3.7 Inflammatory Indicators in the Serum of Obese Mice

**FIGURE 5:**
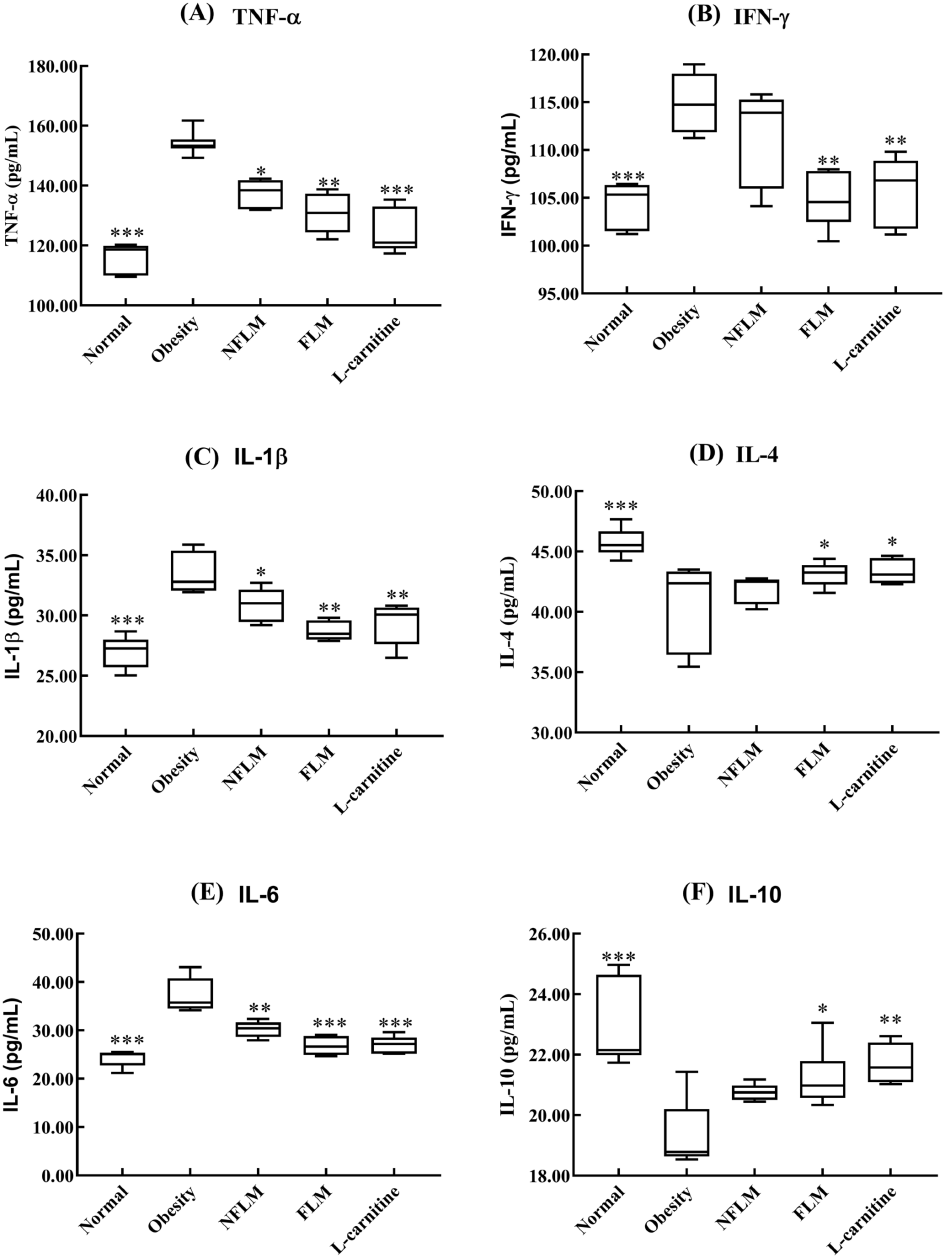
Inflammatory indicators in the serum of obese mice. (A–F) indicate TNF‐α, IFN‐γ, IL‐1β, IL‐4, IL‐6, and IL‐10, respectively. NFLM, non‐fermented lemon peel filtrate group; FLM, LMSS4‐fermented lemon peel filtrate group. The data were expressed as mean ± standard deviation ($\bar{x}$ ± SD). Compared with Obesity group, *p < 0.5, **p < 0.01, ***p < 0.001.

Chronic inflammation is a phenotype associated with obesity. As shown in Figure 5, the mice in the obese group showed the highest serum levels of TNF‐α, IFN‐γ, IL‐1β, and IL‐6, but the lowest levels of IL‐4 and IL‐10, which differed significantly from the normal group. However, treatment with fermented lemon peel filtrate significantly reduced the serum levels of TNF‐α, IFN‐γ, IL‐1β, and IL‐6, and increased the concentrations of IL‐4 and IL‐6. The results demonstrate that fermented lemon peel filtrate prevents chronic inflammation induced by obesity.

### 3.8 Transcription of Inflammatory Genes

**FIGURE 6:**
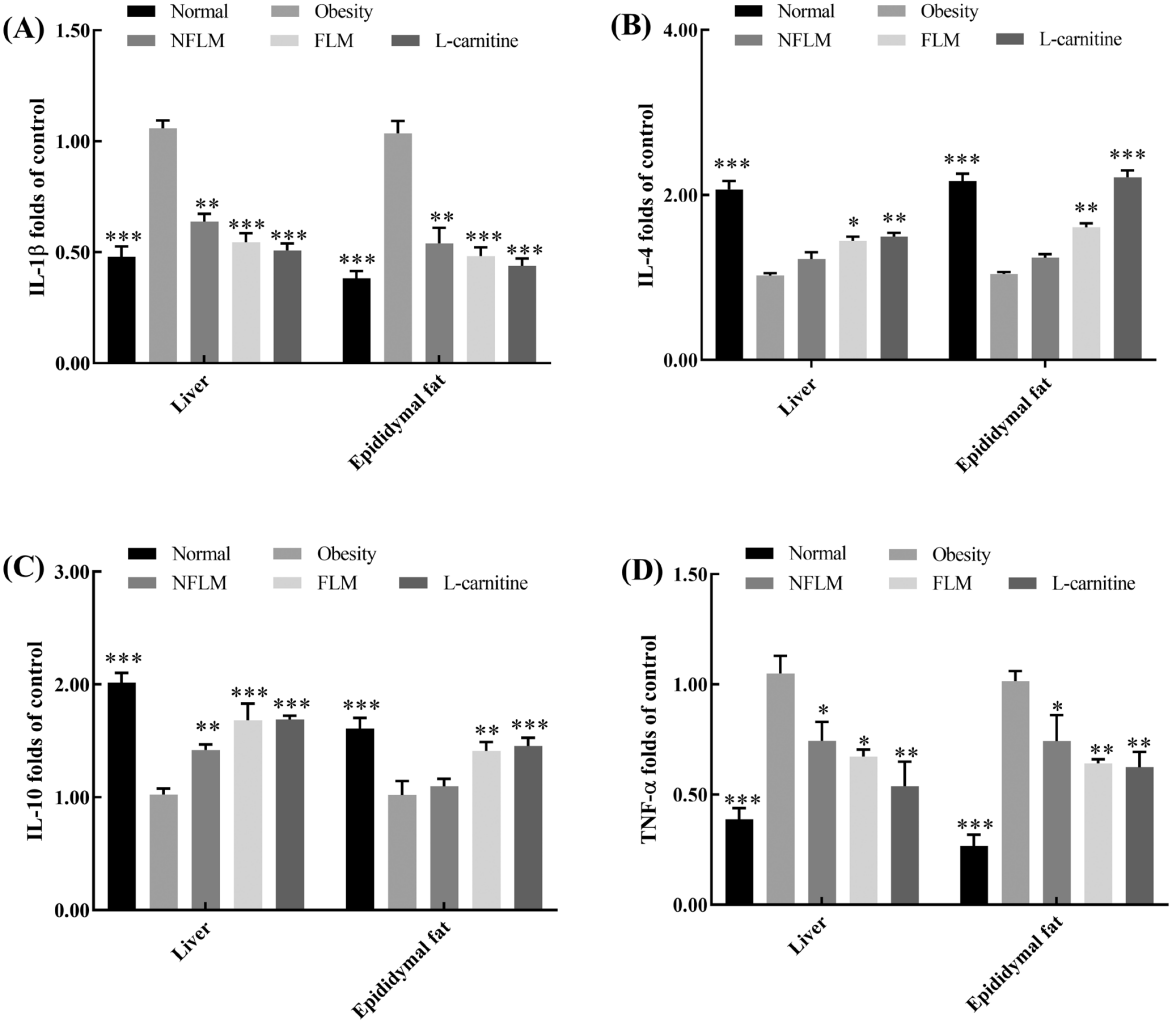
mRNA expression levels of inflammation‐related genes in liver and epididymal fat. (A–D) indicate IL‐1β, IL‐4, IL10, and TNF‐α, respectively. NFLM, non‐fermented lemon peel filtrate group; FLM, LMSS4‐fermented lemon peel filtrate group. The data were expressed as mean ± standard deviation ($\bar{x}$ ± SD). Compared with Obesity group, *p < 0.5, **p < 0.01, ***p < 0.001.

To further evaluate the anti‐inflammatory effect of fermented lemon peel filtrate on HFD‐induced obese mice, the inflammatory gene expression in the liver and epididymal fat tissues was tested by RT‐qPCR. As shown in Figure 6, HFD increased the mRNA levels of IL‐1β and TNF‐α and decreased the expression of IL‐4 and IL‐10. However, compared with the obese group, fermented lemon peel filtrate significantly enhanced the mRNA expression of IL‐4 and IL‐10, but downregulated the expression of IL‐1β and TNF‐α.

### 3.9 Transcription of Obese‐Related Genes

**FIGURE 7:**
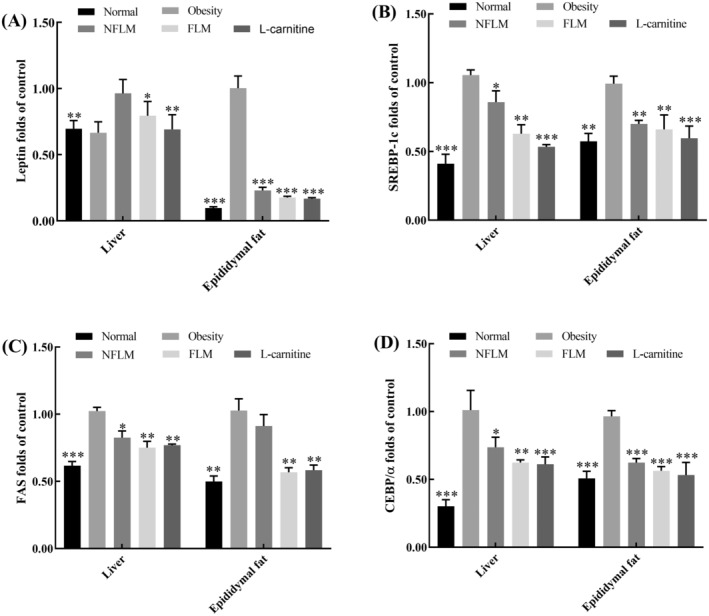
mRNA expression levels of obese‐related genes in liver and epididymal fat. (A–D) indicate Leptin, SRREBP‐1c, FAS, and CEBP/α, respectively. NFLM, non‐fermented lemon peel filtrate group; FLM, LMSS4‐fermented lemon peel filtrate group. The data were expressed as mean ± standard deviation ($\bar{x}$ ± SD). Compared with Obesity group, *p < 0.5, **p < 0.01, ***p < 0.001.

Leptin, FAS, CEBP/α, and SREBP/1c are adipogenesis‐related genes, and obesity may induce abnormal expression of these genes. As shown in Figure 7, mice treated with a high‐fat diet chow expressed high levels of leptin, FAS, CEBP/α, and SREBP/1c mRNA in the liver and epididymal fat tissues. However, the mRNA expression of the above genes was downregulated following oral administration of fermented lemon peel filtrate for 4 weeks. Notably, leptin expression in epididymal tissues was altered most obviously.

### 3.10 Effect of Fermented Lemon Peel Filtrate on NFκB/PPAR‐γ Signaling Pathway

**FIGURE 8:**
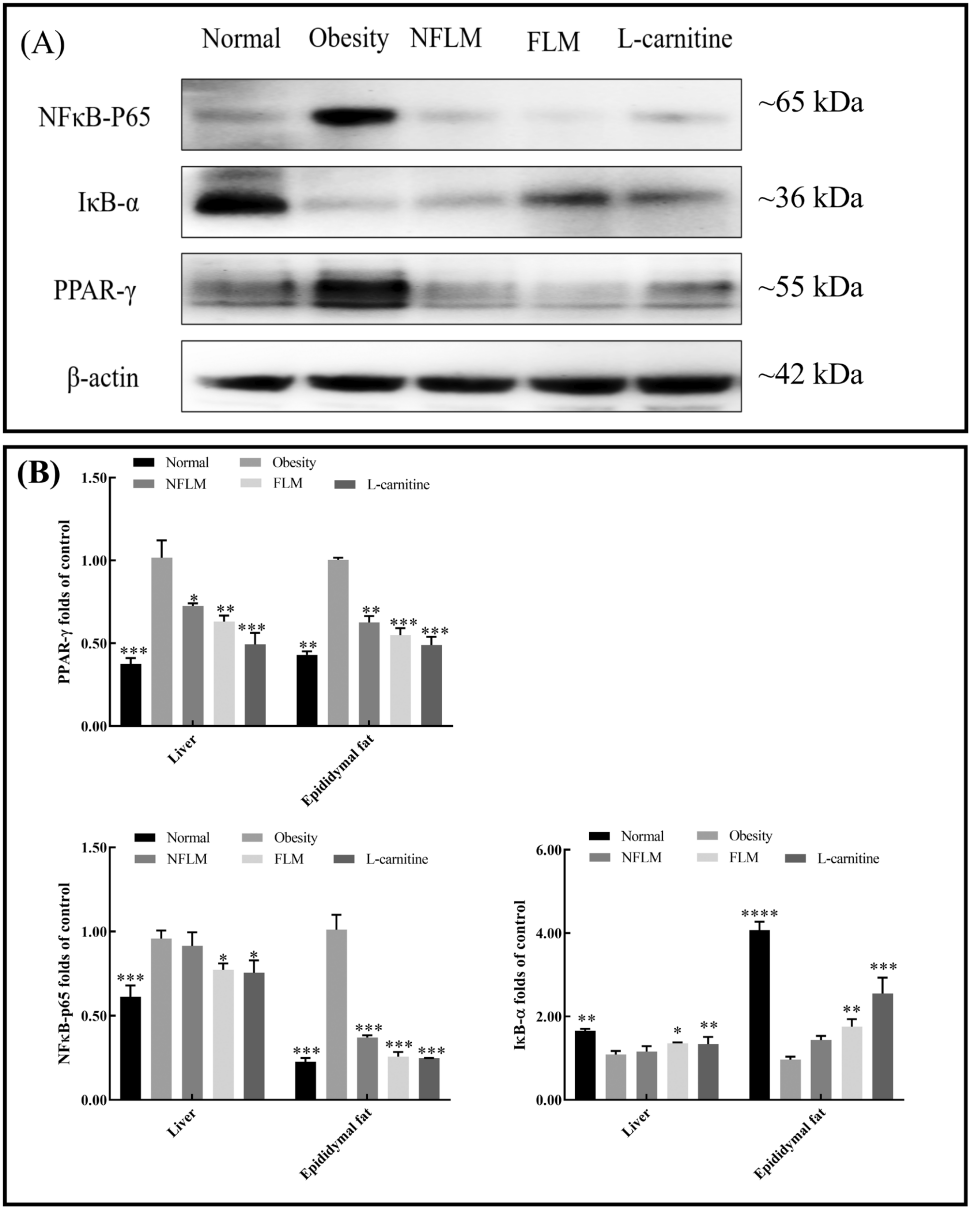
mRNA and protein expression levels of NFκB‐p65, IκB‐α, and PPAR‐γ. (A) Protein expression levels of NFκB‐p65, IκB‐α, and PPAR‐γ in the liver; (B) mRNA expression levels of NFκB‐p65, IκB‐α, and PPAR‐γ in the liver and epididymal. NFLM, non‐fermented lemon peel filtrate group; FLM, LMSS4‐fermented lemon peel filtrate group. The data were expressed as mean ± standard deviation ($\bar{x}$ ± SD). Compared with Obesity group, *p < 0.5, **p < 0.01, ***p < 0.001, ****p < 0.0001.

PPAR‐γ is a crucial regulator of adipocyte differentiation and NFκB plays an important role in inflammation. As shown in Figure 8A,B, the protein and mRNA expression of NFκB‐p65 and PPAR‐γ was upregulated, whereas IκB‐α was downregulated in mice treated with HFD. However, the NFκB‐p65 and PPAR‐γ expression levels decreased, and IκB‐α increased in the NFLM, FLM, and L‐carnitine groups. The expression levels in the FLM group were better than those in the NFLM group and similar to levels in the L‐carnitine and the normal groups.

### 3.11 Constituent Changes Between NFLM and FLM

As shown in Table 3, rutin and hesperidin were the predominant constituents in both the NFLM and FLM among the 15 constituents analyzed. Additionally, scopoletin, ferulic acid, eriocitrin, vitexin, citropten, naringin, cnidicin, neoeriocitrin, and bergaptol were found to be more abundant in the NFLM compared to the FLM. In contrast, chlorogenic acid, umbelliferone, byakangelicin, and oxypeucedanin hydrate were present in the FLM at levels 83.51%, 29.55%, 22.90%, and 30.75% higher, respectively, than those found in the NFLM.

**TABLE 3:** Constituent changes between NFLM and FLM.

Average of Area (mAU*s)					Increased compound after fermentation
NO.	Rt (min)	Compound Name	Before fermentation	After fermentation	
1	7.9	Chlorogenic acid	109.889	201.660	↑(83.51%)
2	12.6	Scopoletin	41.929	21.270	
3	14.4	Umbelliferone	19.874	25.748	↑(29.55%)
4	21.1	Ferulic acid	371.070	12.323	
5	24.8	Vitexin	43.126	39.534	
6	43.2	Citropten	388.183	306.291	
7	24.6	Eriocitrin	3864.128	3590.148	
8	29.6	Naringin	199.983	190.493	
9	32.1	Hesperidin	3256.449	2670.081	
10	38.3	Byakangelicin	191.828	235.764	↑(22.90%)
11	49.2	Cnidicin	47.023	5.701	
12	25.4	Rutin	104.596	88.913	
13	26.8	Neoeriocitrin	202.166	173.367	
14	38.9	Oxypeucedanin hydrate	253.466	331.404	↑(30.75%)
15	45.6	Bergaptol	82.510	51.045	

*Note:* Rt means retention time; Rt is the time of the second parallel experiment. Increase rate (%): [(FLM‐NFLM)/NFLM] × 100. “↑” means increased. FLM: lemon peel fermentation filtrate; NFLM: lemon peel non‐fermentation filtrate.

## 4 Discussion

The present study demonstrated the anti‐obesity effect of LMSS4‐fermented lemon peel filtrate on HFD‐induced obese mice. LMSS4‐fermented lemon peel filtrate inhibited body weight gain, decreased liver and epididymal fat indices, improved the histopathological morphology of liver and epididymal tissues, and maintained serum lipid levels, liver function, and inflammatory indicators at relatively normal levels. Meanwhile, LMSS4‐fermented lemon peel filtrate also regulated obesity‐related gene and protein expression in liver and epididymal tissues. These results suggest that LMSS4‐fermented lemon peel filtrate is a potential anti‐obese substance.

Being overweight is a manifestation of obesity, and weight management reduces obesity‐related complications and improves the quality of life in obese patients (O'Neil et al. 2018). In the present study, we found that LMSS4‐fermented lemon peel filtrate inhibited weight gain in HFD‐induced obese mice. The inhibitory effect of fermented lemon peel filtrate was better than that of unfermented lemon peel filtrate. A previous study reported similar results in that *Lactobacillus plantarum* EM‐fermented cabbage‐apple juice decreased the body weight of HFD‐induced rats (Park et al. 2020).

Prolonged intake of HFD accelerates the accumulation and activation of white adipose in visceral and other tissues, which leads to metabolic abnormalities, such as non‐alcoholic fatty liver, Type 2 diabetes, cardiovascular diseases, and insulin resistance (Cipolletta et al. 2012). Epididymal fat is a white adipose tissue that turns hypertrophic and hyperplastic with continued excess energy intake (Sárvári et al. 2021). Our results indicated that LMSS4‐fermented lemon peel filtrate effectively decreased the liver and epididymal fat indices, and reduced hepatic steatosis, hypertrophy, and hyperplasia of epididymal fat. Our results are consistent with a previous study, which reported that consuming fermented blackberry–blueberry beverages reduced epididymal fat weight, and increased lipid deposition in liver tissues, suggesting the beneficial role of phenolic compounds (Johnson et al. 2016). Furthermore, research indicates that the fermentation products of *Leuconostoc mesenteroides* , including butyrate and other short‐chain fatty acids, can regulate insulin and blood glucose levels in Type 1 diabetes mice, as well as reduce abdominal fat accumulation induced by a high‐fat diet (Pham et al. 2020; Traisaeng et al. 2020). This suggests that in our experiment, the use of *L. mesenteroides* during the fermentation of lemon peels may not only transform the active compounds present in the peels but also produce short‐chain fatty acids through its own fermentation metabolism, which have weight loss effects.

Both animal and clinical studies have reported that obesity is usually associated with higher levels of TG, TC, LDL‐C, and lower levels of HDL‐C compared with those detected in healthy individuals (Park et al. 2021). Probiotics such as *L. rhamnosus* JL1 prevent obesity by reducing the levels of TG, TC, and LDL‐C in HFD‐induced obese mice (Yang et al. 2021). Citrus pectin improves the levels of TG, TC, LDL‐C, and HDL‐C in the blood and ameliorates Type 2 diabetes in rats (Liu et al. 2016). AST, ALT, and AKP are important metabolic enzymes that are secreted into the blood during the necrosis of liver cells (Zhang et al. 2021). In this study, both unfermented and LMSS4‐fermented lemon peel filtrate reduced serum levels of TG, TC, LDL‐C, ALT, AST, and AKP, and enhanced HDL‐C levels. However, fermented lemon peel filtrate was more effective in improving these indicators than unfermented ones, which may be related to the hypoglycemic and hypolipidemic properties of total flavonoids in lemon peel (Kong et al. 2020).

Obesity is usually accompanied by low‐grade inflammation. It is an important phenotype of obesity characterized by increased levels of proinflammatory cytokines, such as TNF‐α, IFN‐γ, IL‐1β, and IL‐6 (Bowers and Singer 2021). Toll‐like receptors (TLRs), especially TLR2 and TRL4, play an important role in obesity‐related inflammation. They are activated by diet‐derived saturated fatty acids, which further increase the receptor‐mediated expression of NFκB, TNF‐α, and IL‐6 (Monteiro and Azevedo 2010). Adipocytes also synthesize and secrete many pro‐inflammatory cytokines such as IL‐1β, IL‐6, TNF‐α, and IFN‐γ (Qatanani and Lazar 2007). Deletion of FAS decreases inflammation in adipose tissues, and FAS mutations increase the expression of IL‐4 and IL‐10 genes to decrease inflammation in adipose tissues (Wueest et al. 2010). In the present study, we found that HFD‐induced obese mice showed higher levels of TNF‐α, IFN‐γ, IL‐1β, and IL‐6 and lower levels of IL‐4 and IL‐10 in serum, liver, and epididymal tissues than the mice exposed to normal fat diet. However, the above indicators appeared to improve after the obese mouse was treated with unfermented and fermented lemon peel filtrates. Citrus flavonoids bind to and decrease the activity of cyclooxygenases (COXs) which are induced in inflammation (Maleki et al. 2019).

Leptin, a hormone involved in energy regulation, is secreted by mature adipocytes accumulating triglycerides. Leptin is overexpressed in obese individuals following the abnormal accumulation of triglycerides, which leads to “leptin resistance” (Montalbano et al. 2019). The sterol regulatory element‐binding proteins (SREBPs) belong to regulatory genes in lipid homeostasis. The mRNA expression of SREBP‐1c in obese mice was increased (Chen et al. 2022b). Meanwhile, FAS and CEBP/α are also lipogenic genes, HFD significantly increased the mRNA expression of FAS and CEBP/α in obese mice (Lee et al. 2019). In the present study, we found that the mRNA expression levels were increased obviously in mice fed with HFD than those fed with a normal‐fat diet. The results also revealed that both unfermented and LMSS4‐fermented lemon peel filtrate downregulated the transcription of leptin, SREBP‐1c, FAS, and CEBP/α in the liver and epididymal tissues of obese mice. Fermented lemon peel filtrate was more effective than unfermented lemon peel filtrate.

In addition to improved expression of lipogenic and inflammatory genes, the unfermented and fermented lemon peel filtrate improved the mRNA and protein expression of key biomarkers in obese mice, such as downregulation of the expression of NFκB‐p65 and PPAR‐γ, and upregulation of IκB‐α. Obesity has been related to the chronic activation of proinflammatory signaling pathways, in which the NFκB is a critical component that controls the transcription and release of downstream pro‐inflammatory cytokines, such as IL‐6, TNF‐α, and IL‐1β (Nisr et al. 2019). IκB‐α is an important inhibitor of activated NFκB, and the phosphorylation of IκB‐α triggers the nuclear translocation of NFκB, and further promotes the release of other cytokines (Singh and Singh 2020). PPAR‐γ is a master transcription gene that induces adipocytic differentiation and adipogenesis. Downregulation of the PPAR‐γ pathway decreases the expression of other adipogenesis genes (such as leptin, SREBP‐1c, FAS, and CEBP/α) and inhibits adipogenesis (Wu et al. 2022). The present study indicates that the anti‐obesity effects of lemon peel filtrates may be related to their inhibition of the activation of NFκB and PPAR‐γ signaling pathways.

The constituents changes between unfermented and LMSS4‐fermented lemon filtrates were analyzed by HPLC. The results showed rutin and hesperidin were the predominant constituents in both the unfermented and LMSS4‐fermented lemon filtrates among the 15 constituents analyzed. In addition, chlorogenic acid, umbelliferone, byakangelicin, and oxypeucedanin hydrate were increased in the fermented lemon filtrate, in which chlorogenic acid showed the highest increase rate (83.51%). However, copoletin, ferulic acid, eriocitrin, vitexin, citropten, naringin, cnidicin, neoeriocitrin, and bergaptol were found to be more abundant in the unfermented lemon filtrate compared to the fermented lemon filtrate. Previous study have verified that rutin and hesperidin could prevent the occurrence and progression of nonalcoholic fatty liver disease (Gong et al. 2024). The umbelliferone, byakangelicin, and oxypeucedanin hydrate were reported to improve diabetic nephropathy (Jin and Chen 2022), murine osteoarthritis (Zhang et al. 2020), and rheumatoid arthritis (Liu et al. 2024). Both chlorogenic acid and ferulic acid are phenolic acids found in star anise, which induced obvious anti‐oxidant and anti‐obesity effects in obese mice exposed to HFD (Iftikhar et al. 2022). The overall findings indicate that the LMSS4‐fermented lemon peel filtrate exhibited higher anti‐obesity effects than non‐fermented filtrate, which may suggest that the increased constituents in fermented lemon filtrate is better than those decreased constituents in preventing obesity.

## 5 Conclusions

The current study revealed that LMSS4‐fermented lemon peel filtrate effectively improved HFD‐induced obesity in mice by regulating the NFκB/PPAR‐γ signaling pathway. HPLC analysis found the rutin and hesperidin were the predominant constituents in both the unfermented and LMSS4‐fermented lemon filtrates, and chlorogenic acid, umbelliferone, byakangelicin, and oxypeucedanin hydrate were increased in the fermented lemon filtrate, in which chlorogenic acid showed the highest increase rate (83.51%). These results suggest that the nutritional value of lemon can be enhanced by fermentation to prevent and treat obesity.

## Author Contributions

**Xianrong Zhou:** conceptualization (equal). **Jia Liu:** conceptualization (equal). **Ruokun Yi:** conceptualization (equal). **Yongpeng He:** conceptualization (equal). **Lujun Chen:** writing – review and editing (equal).

## Conflicts of Interest

The authors declare no conflicts of interest.

## Data Availability

All data generated during this study are available from the corresponding author on reasonable request.
